# Bringing Numbers to Life and Reducing Anxiety: An Augmented Reality and Haptic Feedback-Based Mathematics Game for Primary School Students

**DOI:** 10.12688/f1000research.170339.1

**Published:** 2025-10-17

**Authors:** Andry Ananda Putra Tanggu Mara andryananda.2023, Hilda Widia Rita Hamid, Margaretha Ivana Angeline, Inayah Wulandari, Lutfi Marfuah, Abdul Khair Idham Hamzah

**Affiliations:** 1 Vocational and Technical Education, Yogyakarta State University, Yogyakarta, Indonesia; 2 Faculty of Mathematics and Natural Sciences, Universitas Negeri Yogyakarta, Yogyakarta, Special Region of Yogyakarta, Indonesia

**Keywords:** Augmented Reality; Haptic Feedback; Mathematics Learning; Math Anxiety; Elementary Education; Educational Games; Technology-Enhanced Learning

## Abstract

Mathematics is a basic discipline which causes anxiety to primary school students, resulting in reduced motivation for learning and poor achievements. Traditional instruction heavily leans on abstract symbols, which can add to cognitive load and a feeling of emotional opposition. To address this issue, this study developed a learning model integrating augmented reality (AR) and haptic feedback to create an interactive and multisensory mathematics experience. A quasi-experimental design was conducted with 120 students divided into experimental and control groups. The experimental group used the AR–haptic mathematics game for six weeks, while the control group learned through textbooks and teacher explanations. Data was gathered by using the conceptual understanding tests, a mathematics anxiety scale, classroom observations and student interviews. The quantitative data were analyzed by t-test, ANCOVA and MANOVA analysis; qualitative data were analyzed positionally. The results indicated significant progress in experimental groups’ conceptual understanding and mathematics anxiety decrease when compared to the control group. The mean for the post-test experimental group was 82, and for the control group, it was 62 with a large effect size. The average level of maths anxiety decreased in the experimental group from 3.2 to 1.8 on a five-point scale. Qualitative feedback revealed that students found the game engaging, enjoyable, and less intimidating than traditional lessons. The integration of AR and haptic feedback effectively enhanced both cognitive and affective outcomes in mathematics learning. The multisensory environment enabled students to interact with abstract concepts in tangible ways, reducing anxiety and promoting positive attitudes toward mathematics. This provides a proof of concept for the effectiveness of immersive technologies as assistive tools in the enhancement of academic achievement and emotional health related to elementary level mathematics learning.

## 1. Introduction

The subject “Mathematics” teaches such abstract and logical reasoning as well as problem-solving skills, which are considered to form the basic of lifelong learning. Yet for many elementary school students, math is gets associated with feelings of anxiety provoking a very real, genuine fear that can have long-lasting effects on their academic success. As for other negative outcomes on math understanding, math anxiety hinders the abilities of students to gain conceptual understanding and to be involved in a full range of learning activities ( Erbaş & Demirer, 2025). Tackling this twofold problem, two ways to increase mastery and liking of mathematics appear necessary: improving cognitive mastery and affective engagement with mathematical content. In fact, systematic reviews have demonstrated that children’s math anxiety is a common issue worldwide and we need evidence-based interventions combining pedagogy and technology ( Balt, Börnert-Ringleb, & Orbach, 2022; Ersozlu, 2024).

Mathematics anxiety among primary school students is common, and continues at alarming rates. A previous study conducted in Banda Aceh showed that the mean percentage score of students with math anxiety was 62.58%, most of whom were classified into very high or high categories ( Jannah, Halim, & Asmara, 2024). In the same vein, a study carried out during COVID-19 showed that most of the students experienced moderate levels of maths anxiety as well, which was also markedly disruptive to students’ mathematics learning comfort and effectiveness ( Sugiarti & Kusmayanti, 2022). This vertigo directly affects your numeracy. For example, Nur Awal (2024) noted that mathemath anxiety is greatly to hinder the numeracy literacy of elementary students so this would be difficult for them understand and solve number problem. Elsewhere, Mangkuwibawa et al. (2023) reported a negative correlation between math anxiety and confidence and accuracy in solving numerical problems, with high anxiousness impairing student performance by as much as 21.71%. There are also extra factors that contribute to this challenge from outside. The study in Pacitan found that students had difficulty grasping mathematical instructions, disliked maths lessons and manifested anxiety and physical distress while dealing with maths activities. Factors that could contribute to this is poor mastery in concept, teaching method which was not effective, lack of motivation and boredom on learning ( Putra, 2024). These results are consistent with contemporary meta-analyses that suggest the classroom learning environment, pedagogy and social emotional support feature prominently in math anxiety ( Balt et al., 2022; O’Hara et al., 2022).

With the development of technology, more and more innovative tools are being used to enhance the learning experience. Out of all these, AR has emerged as a concept that concretizes the abstract concepts into interactive 3-dimensional visualizations. As documented in these studies, AR enhances the geometry ability and meanwhile reduces cognitive load and increases students’ flow experience for elementary school students ( Li et al., 2024; Wu et al., 2024). Quit distinctively, AR math space is proven as a creativity induction in primary classrooms (Chen et al., 2019; Hanggara, Qohar, & Sukoriyanto, 2024). Meta-analyses also support the notion that AR enriches student motivations and learning achievements provided it is integrated exploiting strong pedagogical designs (Ahmad & Junaini, 2019; Garzón, Kinshuk, Baldiris, Gutiérrez & Pavón, 2020; Bulut & Ferri, 2023). Moreover, contemporary studies also shed light on the significance of culturally responsive uses of AR and VR for teaching mathematics to effectively reach diverse populations ( Bertrand, Sezer, & Namukasa, 2024). Meanwhile, haptic feedback offers a tactile response that enhances the multisensory learning. The sense of touch is actively engaged by haptic interaction, leading to more conceptual understanding, motivation and collaboration in education (Hatira & Sarac, 2014). Embodied designs as systems for mathematics learning Understood in the above way, embodied design for the teaching and learning of mathematics can have many of its features seen as part of a system—or rather, it would be an expense to emphasize further this sense that an embodieoned design or classroom is a system. New developments now even demonstrate how a combined AR-haptic system can handle fine motor and precision activities, which make haptics in education promising ( Hamad et al., 2025).

However, the combination of AR and haptic feedback in mathematics study for children was rarely studied, also limited to early age education. The bulk of previous research has studied AR in a standalone fashion and its effect on academic performance ( Li et al., 2024; Chen et al., 2019) or motivation ( Debrenti, 2024), while the literature with regard to haptics has been limited to laboratory-based or prototype studies ( Hatira & Sarac, 2024). The potential for integrating AR and haptic feedback to address cognitive as well as affective dimensions—specifically math anxiety—has not been adequately analyzed ( Moreno et al., 2023; Jiang, Zhu, Chugh, Turnbull, & Jin, 2025).

Thus, the proposed methodology in this study is referred to as “Bringing Numbers to Life and Reducing Anxiety: An Augmented Reality and Haptic Feedback Based Game for Elementary School Students”. Unlike the traditional teaching materials, where students are expected to remember human structure from abstract symbols by rote, perception of spatial relation is developed on a multisensory learning environment of virtual and immersive technologies. By uniting the visual affordances of AR with the tactile-engagement afforded by haptic feedback, the game intends to turn learning mathematics into an embodied, interactive, emotionally supportive experience. One primary purpose of this research is to improve students’ conceptual knowledge and understanding in mathematics, as well as meeting their affective needs such as decreasing math fear or anxiety. Through three-dimensional manipulation and observation of the mathematical objects in AR environment, their conceptual understanding is anticipated to be enhanced by closing the gap between abstract symbols and concrete representations ( Li et al., 2024; Ahmad & Junaini, 2020). Furthermore, haptic feedback delivers direct tactile responses to students, correlating study with one’s hands and promoting the sensation of presence (and engagement) (Hatirassa & Saracss, 2024).

Additionally, the simultaneous integration is used to not just enhance cognitive achievements, but also develop appropriate emotional involvements. The working memory and problem-solving efficiency have been disrupted by the MA ( Erbaş & Demirer, 2025) and most of these interventions are pedagogical or counseling based ( Balt et al., 2022). However, the potential of immersive digital environments for relieving this anxiety has so far gone largely unnoticed. What the game does is incorporate a haptic response into AR based learning activities, which introduces calming rewards and confidence building feedback that may suppress negative affective reaction or build (math resilience) activism in math ( Canright & White Brahmia, 2024).

In doing so, the contribution of this paper rises above previous literature in three important ways:

a. It combines AR and haptic feedback in one pedagogical design, an intersection that has been explored little in elementary mathematics education ( Crandall & Karadoğan, 2021; Jiang et al., 2025).
b. Addresses both cognitive (conceptual understanding) and affective outcomes (reducing math anxiety) directly instead of in isolation ( Balt et al., 2022; Schoenherr, Schukajlow, & Pekrun, 2025).
c. It contributes to the development of embodied learning theory by actualizing multisensory interaction in a real classroom setting and opens up new directions for technology enhanced learning research and practice ( Bertrand et al., 2024; Garzón et al., 2020).

This research provides the following contributions to technology-enhanced learning: (1) combining AR and haptic feedback into a single mathematics game developed for elementary school students, (2) targeting both cognitive outcomes and affective issues such as math anxiety, and (3) operationalizing the principles of embodied design in a game-based learning environment to promote sustainable, emotionally positive learning of mathematics.

## 2. Methodology

### 2.1 Research design

**Supplementary Figure 1.** Plan and Research Design

In this case we applied the quasi-experimental pre-test/post-test control group design as shown at supplementary figure 1, a highly respected approach in educational technology research ( Creswell & Guetterman, 2019; Ary et al., 2020). The mixed-methods approach employed allowed for the fusion of quantitave data around learning gains and math anxiety with qualitative analysis focused on students’ perception of the experience in order to provide a nuanced description of effectiveness ( Johnson & Onwuegbuzie, 2020). This design also aligns with frameworks stating that interventions for math anxiety include an affective and cognitive measurement to assess the effects of digital game-based approaches ( Kahveci, 2022; Lagos San Martín, Ossa, Kaur Swaran Singh, & Mahmud, 2023). A diagrammatic representation of the mixed-method design incorporating quantitative and qualitative lines of inquiry is presented in Figure. The study starts with a pre-test through CUT and MARS-E to the two groups. After that, the intervention phase is implemented in which the experimental group uses an AR haptic game and control group is given traditional teaching. At the end of the intervention, both groups take a post-test with the same scales in order to measure changes and learning results. Results of the post-test measures are then examined to determine experiment effects, specifically within cognitive and affective domains. Simultaneously, the qualitative strand is performed to supplement results. This follows interviews and a teacher FGD to gain further insights into perceptions, experiences, guidance surrounding the interventions. The quantitative results and qualitative findings are then combined at the integrated findings stage, providing a more holistic understanding of the results, which connect numerical changes to experiential insights. This approach guarantees a nuanced analysis, which includes both how the intervention worked and what each study participant had experienced.

### 2.2 Participants

The sample consisted of 120 elementary school students (ages 9–11 years) drawn from two public schools in Yogyakarta. To ensure compliance with research ethics and educational regulations, the study was formally approved by both the research institution and the Department of Education and Youth Affairs, Yogyakarta City Government. Official research permission was granted, and the authorization letter is publicly accessible via its DOI link: https://doi.org/10.5281/zenodo.17120705 *.* Balanced representation was ensured by applying stratified random sampling ( Etikan & Bala, 2017). Half of the students joined one group:

a. Experiment group (N = 60): were taught mathematics using an AR and haptic feedback game.
b. Control group (n = 60): students were taught mathematics through textbooks and teachers’ explanations in classes.

All students shared the same prior exposure to mathematics according to baseline tests, consistent with sampling recommendations in experimental educational research (Fraenkel et al., 2019). It is demonstrated in earlier research that math anxiety rates among children of this age typically associate with gender disparities, previous performance, and learning atmosphere ( Mitchell & George, 2022; Möhring, Moll, & Szubielska, 2024). This paper investigates the relationship between young Polish elementary schoolchildren’s self-reported mathematics anxiety and their actual numerical achievement. It was thus essential to make comparability of groups to minimize extrinsic bias.

### 2.3 Intervention design

The intervention was of six weeks duration, twice a week for 45 minutes. The game, “Bringing Numbers to Life”, was developed in Unity 3D, incorporating AR markers and haptic glove devices as an example of contemporary immersive learning design ( Radu, 2021; Santos et al., 2020). Its key features included:

a. AR Visualization – improving representation of fractions, geometry and multiplication ( Akçayır & Akçayır, 2022; Kaźmierczak et al., Skowroński, Kaźmierczak, & Kowalczyk -Page 8-572; Grunwald 2025).
b. Haptic Feedback Integration— to allow kinesthetic interaction, which is known to enhance memorisation, engagement and learning for sighted ( Wang et al., 2021) and visually impaired children ( Marichal, Rosales, González Perilli, Pires, & Blat, 2023; Mutis & Oberemok, 2025).
c. Game Design Mechanics – utilising challenges and rewards to create motivation ( Koivisto & Hamari, 2019).

Two weeks of training were provided to the teachers prior to implementation, which is aligned with guidance regarding fidelity when implementing technology-enhanced classrooms ( Bower, 2019). Further, it was found that the application of immersive AR and haptic experiences to tackle academic and emmotional barriers of learning (supported by sustainability-focused pedagogies for future ready education) such as those postulated by ( Mirza, Dutta, Tuli & Mantri, 2025), were clearly conceptualized.

### 2.4 Instruments

a. Conceptual Understanding Test (CUT): A 25-item test Verified through review by experts (Cronbach’s α = .86).
b. Mathematics Anxiety Rating Scale for Elementary students (MARS-E): A version tailored to be context-local, with satisfactory reliability (α = .91), based on existing math anxiety measures ( Carey et al., 2019). These are important points to consider as anxiety and attainment in Mathematics have a strong relationship ( Möhring et al., 2024).
c. Observation Protocol: Informed by classroom context models of engagement ( Fredricks, Blumenfeld, & Paris, 2016), with additional attention to how instruction and the social climate of classrooms may mediate a reduction in anxiety ( O’Hara, Kennedy, Naoufal, & Montreuil, 2022).
d. Student interviews: Semi-structured interviews to collect affective and experiential feedback; commonly found in AR-based learning papers ( Ibáñez & Delgado-Kloos, 2018).

### 2.5 Data collection procedures

The research proceeded in three phases:

a. **Pre-test Phase:** Both groups completed CUT and MARS-E.
b. **Intervention Phase:** Experimental group engaged with the AR-haptic game, while the control group used conventional methods. Observations and interim reflections were recorded ( Miles, Huberman, & Saldaña, 2020). The design explicitly embedded game-based learning principles shown to reduce math anxiety and foster motivation ( Kahveci, 2022; Pahmi et al., 2023).
c. **Post-test Phase:** Both groups completed CUT and MARS-E again, followed by interviews and teacher focus groups.

### 2.6 Data analysis

Quantitative analysis was performed on SPSS 29. Group differences were analyzed using independent samples t-tests, paired t-test and ANCOVA as recommended in the best pratenice approach of educational experimental research ( Field, 2021). Modifier variables were control for and effect sizes (Cohen’s d) were computed to assess practical significance. MANOVA was performed to detect multidimensional changes for anxiety data. These analyses are consistent with suggestions that math anxiety be inspected in both cognitive and affective space ( Pizzie & Kraemer, 2023; Polydoros et al., 2025).

Thematic analysis of qualitative data was conducted using NVivo 14 according to Braun and Clarke’s (2019) six stages of thematic analysis. Themes were related to student engagement, motivation and connectedness, with an AR integration that have been emphasized in previous game-based learning and AR integration studies ( Lagos San Martín et al., 2023). The application of triangulation between test findings, observations, and interviews served to strengthen the reliability of results ( Lincoln & Guba, 2018).

### 2.7 Ethical considerations

This study was conducted in accordance with institutional ethical standards for research involving human participants ( BERA, 2018). Prior to data collection, ethical approval was obtained from the Research Ethics Committee of Yogyakarta State University with letter number: B/3018/UN34.13/TU.01/2025, and official permission was granted by the Yogyakarta City Education and Youth Office with letter number: 000.9/1581 (Tanggu Mara. A., 2025). As the children were in primary school (9–11 years), the written consent was sought from both the parents/guardians of students and school authorities. Moreover, the children also gave their verbal assent prior to participation and it was explained to the children in an age-appropriate manner thatparticipation was voluntarily, they could withdraw from the study at any time. All recruited subjects were briefed on this, and told that these data will be kept confidential, anonymous, and for academic use only. Also, they were informed that in case of non-agreement and withdrawal at any time there would not be any prejudice. Verbal assent, as well as written parental consent, was utilised to verify understanding and comfort of the children participants in all aspects of the research ( Mitchell & George, 2022; O’Harra et al., 2022).

## 3. Results and discussion

### 3.1 Game prototype development outcomes

The prototype of AR and haptic feedback-based math game effectively combined three key components, which are the interactive AR objects, problem-solving missions within contexts embedded storylines, and the tactile response that emulates reality in contact with numbers. This set up allowed concepts from abstract math theory (place value, adding, subtracting and multiplying) to come alive as tangible interfacing. Evaluation of the prototype system by instructional designers and teachers indicated its compliance with usability, accessibility requirements, as well as its relationship to pedagogical aspects in primary classroom. These results are in a line with Garzón et al. (2020) and Ahmad et al. (2020), whose reviews suggest that AR interventions improve engagement and results when underpinned by pedagogy. In addition, the provision of haptic feedback is a relatively new factor learning mathematics. Touching numbers with the game provided a bridge between abstract symbols and sensory input, re-emphasizing the kinesthetic learning pathways ( Crandall & Karadoğan, 2021; Hatira & Sarac, 2024). Consistent with observations of Marichal et al. (2023), haptic feedback was particularly beneficial for learners who needed multimodal input. This addresses previously professed standards in mathematics education research to create embodied experiences for children who struggle with abstract ways of thinking (Nemirovsky et al., 2020; Bertrand, Sezer, & Namukasa, 2024).

The developed maths game app is available at https://kidar-playtime.lovable.app/ and serves as the central intervention of this research. The app combines Augmented Reality (AR) visualization with haptic feedback to create an augmented multisensory space for learners to interact with mathematical ideas. Before final release, the game was reviewed by domain experts Nsoccer systems, instructional design and maths who checked for content accuracy and its pedagogical integrity. Al-i-Imran (240), 50 Malay and nine hadith verses on head reduction of disease for six months. The resulting application was trialed with samples, which could prove the technical reliability and pedagogical impact. Comparable validation procedures have been documented in AR learning-related research to confirm the implementation fidelity ( Hanggara, Qohar, & Sukoriyanto, 2024; Pahmi et al., 2023).

**Supplementary Figure 2.** User Interface of AR Game

The user interface and interactive features of the AR-based haptic mathematics game are shown in supplementary figures 2. The design is centered on facilitating accessibility with simple and easy-to-understand controls, visual feedback, and tactile experiences to provide students exposure to “feeling” the basics of arithmetic. This multimodal approach not only promotes conceptual understanding, it also develops positive affective response, which is consistent with evidence suggesting that technology-rich learning environments can alleviate obstacles like math anxiety and promote engagement (Wu et al., 2022; Fidan & Tuncel, 2019).

Table 1 Descriptive statistics of the research results for control and experimental groups. A trend of development was also found for the control group, even if very small (Mean = 58, s.d. = 10 pre-test; Mean = 62, s.d. = 9 post-test). In contrast, the experimental group demonstrated a marked improvement from pre-test (Mean = 59, SD = 11) to post-test (Mean = 82, SD = 8), indicating that treatment effects were large for participants in this condition.

**Table 1.** The result statistic of control and experimental Pre-Post test.

Group	N	Mean	SD
Control Pre-test	60	58	10
Control Post-test	60	62	9
Experimental Pre-test	60	59	11
Experimental Post-test	60	82	8

Table 2 presents the statistical tests that we have done to analyze these differences. Those in the control group improved, pre-test versus post-test (Mean Difference = 4) was significant statistically (p = 0.08), demonstrating that without intervention, performance increased barely at all. There was significant difference between experimental and control group after the pretest level (Mean Difference = 23, p = 0.001), indicating that the intervention had an impact on the team members’ learning achievement. Also, in the comparison of post-test means between experimental and control groups, being experimental group adopts more superior than that of the control group (Mean Difference = 20, p = 0.001) with large effect size (Cohen’s d = 2.35), supporting the powerful impact of intervention.

**Table 2.** Comparison of control and experimental tests.

Comparison	Mean difference	p-value	Cohen’s d
Control (Post - Pre)	4	0.08	nan
Experimental (Post - Pre)	23	0.001	nan
Experimental vs Control (Post-test)	20	0.001	2.35

This result suggests the potential of combining AR and haptic feedback for improving not only students’ conceptual understanding, but also their motivation and involvement in learning mathematics (Sáez-López et al., 2023). The AR tool was capable for making abstract mathematical conceptations, such as fractions and geometry, to be visualized in a 3D environment interactionally meanwhile the haptic feedback factor made tactile reinforcement who strenghtened the cognitive processes (Shu et al., 2022). The post-test score of the experimental group not only indicates better achievement but also higher levels of attitudinal engagement, which may indicate lower math anxiety (Ramirez et al., 2018; Erbaş & Demirer, 2025). In addition, qualitative feedback obtained in student interviews suggested that students enjoyed playing the game and found it motivating and less threatening than conventional approaches to learning. A number of students wrote that they “were no longer as afraid of making mistakes” and that “math became more fun when the numbers came alive.” This is in line with earlier research which have underscored that game-based learning employing immersive tech nologies can mitigate the pattern of negative emotions and the boost in academic selfconfidence in mathematics learning (Fokides & Tsolakidis, 2022).

### 3.2 Effects on cognitive achievement

Table 3 displays the descriptive statistics of the scores on the CUT and MARS–E for experimental and control groups at pre and post tests. Conceptual understanding the two groups scored almost at a similar level in conceptual understanding at the beginning of the experiment with: Experimental group means score = 53.25, SD = 7.25; control group means score = 54.43, SD = 8.36 (Appendix b). Levels of mathematics anxiety were likewise approaching, with the experimental group having a mean of 66.48 (SD = 7.54) and for control group this was 66.70 (SD = 6.07). The fact that these numbers are comparable in both groups before the application of the intervention. Noticeable differences did arise afterward, however. The AR-haptic game experimental group showed highly significant improvement on conceptual understanding, with a mean of 73.22 (SD = 9.25), compared to the control group that used conventional methods (M1C¼62.43, SD1C¼9.42). The pattern was the same for mathematics anxiety. The experimental condition showed substantial improvements, with a post-test mean of 38.73 (SD = 8.76) as compared to the control’s mean 56.25 (SD = 8.32) little improvement was shown by the controls across assessments. Collectively, these findings suggest that the AR-haptic game intervention was effective in increasing students’ conceptual understanding and reducing their levels of MA compared to traditional teaching.

**Table 3.** Pre-Post CUT and MARS.

Group	Pre_CUT		Pre_MARS		Post_CUT		Post_MARS	
	mean	std	mean	std	mean	std	mean	std
Eksperimen	53.25	7.25	66.48	7.54	73.22	9.25	38.73	8.76
Kontrol	54.43	8.36	66.7	6.07	62.43	9.42	56.25	8.32

**Supplementary Figure 3.** Pre-Post CUT and MARS Treatment

Supplementary Figure 3 Explain there were statistically significant differences between their pre- and post-test results in the experimental group, compared with the control group. Students who were exposed to the AR-haptic game showed larger improvements in number sense, problem-solving accuracy and retention of arithmetic operations than students who did not play consequently. These results are consistent with Cai et al. (2022) and Wu et al. (2024) emphasized those AR enables deeper conceptual understanding by making visible abstract concept and enable manipulation to be part of representation. The tactile manipulations potentiated this effect, consistent with Pila et al. (2020) and Mutis and Oberemok (2025), who demonstrated that haptic-enabled tablets advanced preschoolers’ STEM learning, and underscored the taxonomical role of haptics in education. Moreover, Kaźmierczak et al. (2025) observed similar enhanced cognitive and non-cognitive effects, which assured our findings. Interestingly, the haptic aspect increased cognitive effect by involving multiple sensory modalities. Previous cognitive psychology studies show that learning under the condition of multi-sensory environment enhances information processing and memory retention (Shams & Seitz, 2008; Zhang et al., 2023). In the present study, haptic reinforcement guaranteed learning was not only visual or auditory but rather embodied for durable and deeper knowledge of mathematical representations. This is particularly important in the case of younger students who stand to gain from a hands-on, experiential approach towards teach (Montessori, 2021).

### 3.3 Effects on affective outcomes (Math anxiety reduction)

One of the most surprising findings was that math anxiety decreased for the participants. The survey results revealed that students who had previously feared or avoided mathematics showed increased levels of confidence and fun after having played the AR-haptic game. They also referred to mathematics as “fun” and “less scary,” as they could mess with numbers in a playful setting. This is in agreement with Balt et al. (2022), Sammallahti et al. (2023) and Schoenherr, Schukajlow, Pekrun (2025), who argonued intervention promoting emotonal and cognitive coping together are superior in terms of reducing anxiety. AR was immersive and game-like, so it provided a safe environment to make mistakes learned from the failure as an exploration; similar to that of Kahveci (2022) and Lagos San Martín et al. (2023). Crucially, haptic feedback conveyed immediate tactile sensations that supported reassurance and confidence having been reported also in Yu et al. (2023) in which the AR had a positive effect on anxiety of learning physics via cognitive load reduction.

Psychologically, this illustrates the way in which embodied interactions can overcome affective hurdles. Mathematics anxiety has been associated with cognitive load and self-concept (Ashcraft & Krause, 2007). Through redistribution of cognitive load across sensory channels and supportive tactile cues, the AR-haptic method reduced both the cognitive and emotional demands for problem-solving.

**Supplementary Figure 4.** Anxiety Changes Level After Treatment

Supplementary Figure 4 illustrates the relative increases or decreases in mathematics anxiety across control and experimental groups from pre-test to post-test. Only minimal decreases in math anxiety were found in the control group who reported a mean average score reduction from 3.1 to 2.9 on a Likert scale of agreement of one to five within session scores. This small decrease is likely small as it suggests that traditional methods of instruction were not able to adequately reduce negative affect related to mathematics learning. On the other hand, participants in the experimental group who played with an Augmented Reality (AR), Haptic Feedback mathematics game had a significant reduction on math anxiety from 3.2 to 1.8. This decline reflects that the users felt more engaging, less intimidating learning environment when using AR with multisensory haptic feedback. There was an increased “can do” attitude, enthusiasm and emotional positivity among students toward mathematical tasks. This result supports previous results that technology-based interventions can successfully reduce math anxiety by increasing engagement and reducing cognitive load (Chang et al., 2020; Fidan & Tuncel, 2019). Furthermore, haptic feedback gave the learners a sense of touch which provided them with a multi-sensory experience (Montgomerie et al., 2012) allowing teaching concepts in an abstract way but learning in a concrete manner and reduced affect barriers to learning (Wu et al., 2021), (Ramirez et al., 2018).

As can be seen in Table 4, there is a clear contrast between the two groups (control versus experimental) in terms of mathematics achievement and levels of math anxiety. Overtesting (F = 8.51, p = .**) and the control group evidence only minimal adiective gains from pre- to post-test (F = 4.90^ == > ^p =. 03, students in the experimental group (AR-Haptic math game) demonstrated a significantly higher level of achievement (F = 178.001, η ^2^ = .14). This result implies that immersed MSM interaction by using AR and haptic may provide a significant advantage in promoting the conceptual understanding of students’ learning performance compared with that of traditional teaching.

**Table 4.** Tests of simple main effects — Mathematics achievement and anxiety.

Sources	SS	df	MS	F	p	η ^2^	Post hoc
**Math Achievement**							
Control	102.25	1	102.25	4.90	.03 *	.12	Slight improvement
Experimental (AR-Haptic)	2,081.20	1	2,081.20	178.03	< .001 **	.14	AR-Haptic > Control
Error	—	39	—	—	—	—	
**Math Anxiety**							
Control	472.19	1	472.19	29.36	< .001 **	.09	Minimal reduction
Experimental (AR-Haptic)	1,574.58	1	1,574.58	44.06	< .001 **	.11	Anxiety significantly reduced
Error	—	38	—	—	—	—	

Note: AR-Haptic = Augmented Reality and Haptic Feedback-based Game; MS = Mean square; SS = Sum of squares.

* p < .05;

** p < .001.

With regard to math anxiety, the control group reported a small but significant decrease (F = 29.36, p < .001, η ^2^ = .09). The experimental group, however, showed a greater and significant reduction in anxiety (F = 44.06, p < .001, η ^2^ = .11). This showed that the typtactile approach in which multisensory and tactile engagement was integrated with visual learning, as compare to visuonly helped not only cognitive achievement but also emotional aspects making mathematics learning more favourable and fun environment.

### 3.4 Integrating cognitive and affective dimensions

**Supplementary Figure 5.** Result of Paired T-Test

Supplementary Figure 5 show the Paired T-Test radar chart sheds further light on this comparison, as it represents the results of paired samples t-tests performed for the experiments and controls at each measurement (CUT and MARS-E). Experimental group placement on the graph also demonstrates a significant increase in CUT scores, indicating much increased conceptual understanding post intervention. Meanwhile, the treatment group exhibits also a large change in MARS-E, as an overall decrease of scores reflects a significant decrease in math anxiety. The control group, on the other hand, plots significantly closer to the negative end of both CUT and MARS-E which demonstrates that although there were small increases in conceptual understanding and small decreases in anxiety, they were not as strong as those variables seen with the experimental group. Collectively, the visualization highlights the more pronounced combined impact of an AR-haptic game intervention on learning and anxiety in contrast to the relatively weaker effect from traditional teaching methods.

The double effect of improving cognitive benefits and decreasing affective obstacles, highlights the overall potential of AR and haptics in mathematics education. Previous research typically explored these two dimensions in isolation of one another, looking at either learning gains ( Ahmad & Junaini, 2020; Debrenti, 2024) or anxiety decrease ( Mitchell & George, 2022; Möhring et al., 2024). This manuscript shows that these two challenges could be solved in one shot through visual reinforcement with tactile cues and this is the main contribution of this work. By “making numbers real,” the intervention connected abstract knowledge with lived experience. By “alleviating anxiety,” it paved the way for students to approach mathematics learning emotionally and cognitively unfettered by fear. This pairing underscores an emergent pedagogical direction—designing multisensory and affect-aware educational technologies that is different from the traditional e-learning platforms ( Bulut & Ferri, 2023; Volioti et al., 2023). They also have implications for curriculum concordance. If AR-haptic systems are mainstreamed in classroom instruction, these could possibly function as not only supplementary but central pedagogical tools to transform mathematics learning and teaching ( Mirza et al., 2025; Zapata et al., 2024). This is especially essential in rural/underserved schools where in-service teacher knowledge about math anxiety may not be optimal and such technologies can mediate between affective support and cognitive scaffolding ( Putra, 2024; Ersozlu, 2024).

## 4. Conclusion and implications

### 4.1 Conclusion

The results obtained in Table 5 reveal that the AR-Haptic mathematics game was an overall positive learning experience for students. The majority of participants stated that the game was engaging and pleasant to look at, which increased capacity for capturing attention as well as motivation. Furthermore, the augmented reality combined with haptic feedback supported meaningful understanding through visual elaboration of mathematical concepts which made overall abstract ideas easier to conceptualize. The students also liked the game’s simplicity and its ability to be self-paced so that they could actually go back to play again and reinforce what they had learned. However, some limitations were noticed in this study. Some students had longer trace and response time, and some visual elements were not always easy to follow, which could disturb the interaction conceptual understanding. In order to address such problems, participants had proposed issues like reducing tracking delay time, enhancing visual elements (i.e. fonts, objects) as well as adding additional interactive features to increase attractiveness and usability offer participants of this study all held different opinions on web3D usability according to their pervious experience with web3D transactions and activities. The study shows however that AR combined with haptic feedback has distinctive cognitive and affective effects on mathematics learning for elementary school children. The prototype game designed in this study delivered rich and engaging multisensory experiences that instantly enhanced how well students grasped the concepts being studied, their problem-solving skills even remediate math-anxiety an established roadblock to performance in early education.

**Table 5.** Students’ experience toward the AR-Haptic mathematics game.

Codes	Frequency
Benefits	
Engaging and eye-catching	28
Supports conceptual understanding (visual elaboration)	21
Easy and intuitive to operate	19
Allows self-paced learning and review	16
Drawbacks	
Longer tracking and response time	8
Some visual elements unclear	7
Recommendations	
Reduce tracking time	7
Use larger and clearer visual elements (e.g., fonts, objects)	6
Add more interactive features	4

Note: AR-Haptic = Augmented Reality and Haptic Feedback-based mathematics game.

The results also support the claim that, in learning environments that facilitate multisensory interaction, students are able to connect with abstract mathematical concepts and make them relevant by grounding them on a tangible foundation, rendering the working process exciting and meaningful. In addition, the affective results show that AR-haptic systems have promise in providing emotionally supportive and motivating materials for students to engage with mathematics without fear. Thus, the work emphasises technology’s two-fold function as not only a mechanism to improve academic achievement but also as an emotionally and psychologically supportive education tool.

*“I found the interface very intuitive; I did not need extra guidance to operate the features.”* – **Aisyah Nurul Kharomah**

*“It would be more engaging if additional interactive features were included.”* – **Alya Khairani Mumtaz**

### 4.2 Theoretical implications

Theoretically, this study contributes to discussions of embodied, affect-aware technologies for learning. Previous research has mainly concentrated on either the cognitive or affective aspect of AR teaching and learning (Garzón & Acevedo, 2021; Cai et al., 2022). Augmented Reality for Education: A Multimethod Literature Review but hardly two at the same time. By integrating AR visualization with haptic feedback, the current work shows how learning technologies can scaffold both cognitive success and overcome affective resistance. This is consistent with, and extends, constructivist and embodied cognition perspectives which propose that mathematics learning is most effective when abstract reasoning has roots in real sensory experience (Nemirovsky et al., 2010; Zhang et al., 2023). In addition, the finding that affective states directly impact academic performance further supports findings in educational psychology of emotions constituting an integral part, rather than a mere addendum, of learning ( Pekrun, 2021).

### 4.3 Practical implications

The contributions of this research are as follows:

a. For Teachers and Schools: Mathematics of AR-haptic games can lay out the resources for teachers, use augmented reality (AR) to equip teachers with tools that support their students’ academic and emotions. In the context of limited teaching resources, such tools offer personalized support when teacher support is not available or adequate.
b. For Curriculum Development: The study encourages the inclusion of multisensory, gamified technology in national and local curriculums, especially for early mathematics education. This fits well with worldwide trends in eLearning transformation and competency-based education.
c. For Policymakers and Developers: The study emphasizes how affordable AR-haptic technologies can help reduce educational disparities in underprivileged regions. Policymakers could focus on investing in such technologies through larger efforts to improve STEM learning and bridge the digital divide, particularly in rural or underserved schools.

### 4.4 Limitations and future research

This study has several limitations, however, and while the findings are encouraging they must be interpreted with caution. First, the sample was restricted to a single school and hence this may limit the generalisation of findings. Second, the intervention was short-term and long-term effects on retention of learning, as well as sustained reduction in anxiety, have yet to be examined. Third, although the current study investigated basic arithmetic, future work could expand on its findings toward other mathematical areas (e.g., geometry, fractions or algebra) where AR-haptic may also be applied. Further research could address the cross-cultural use, scalability of the system in large classes and integration with adaptive learning algorithms. A longitudinal perspective would be useful to investigate how these technologies impact students learning trajectories and affective development over time.

### 4.5 Final remark

At last, the study is another piece of evidence to the emerging technolgoy-enhanced learning which may be cognitively empowering and affective supportive as well. In bringing together AR, haptic feedback and mathematics education this research provides a route for creating learning environments that are both more successful as learning spaces but also more humane. With educators globally looking for innovative ways to enhance STEM educational outcomes, AR-haptic games offer a potential pathway towards both capability and confidence building among children.

## Data Availability

The dataset underlying the research have been deposited in Zenodo and are accessible at: https://doi.org/10.5281/zenodo.17120556 (CC0 license) (Tanggu Mara. A., 2025), include all supplementary files: Supplementary figure 1: Plan and Research Design Supplementary figure 2: User Interface of AR Game Supplementary figure 3: Pre-Post CUT and MARS Treatment Supplementary figure 4: Anxiety Changes Level after Treatment Supplementary figure 5: Result of Paired T-Test Supplementary Table 1: The Result Statistic Supplementary Table 2: Summary of Statistical Tests Supplementary Table 3: Pre-Post CUT and MARS Supplementary Table 4: Tests of Simple Main Effects Mathematics Achievement and Anxiety Supplementary Table 5: Students’ Experience toward the AR-Haptic Mathematics Game (Tanggu Mara. A., 2025).

## References

[ref1] Abrahamson D Lee RG Negrete AG : Coordinating visualizations of polynomial multiplication: Toward an embodied design for mathematics learning. *International Journal of Child-Computer Interaction.* 2020;26:100220. 10.1016/j.ijcci.2020.100220

[ref2] Ahmad NIN Junaini SN : Augmented Reality for Learning Mathematics: A Systematic Literature Review. *Int. J. Emerg. Technol. Learn.* 2020;15(16):106–122. 10.3991/ijet.v15i16.14961

[ref3] Akçayır M Akçayır G : Advantages and challenges associated with augmented reality for education: A systematic review of the literature. *Educ. Res. Rev.* 2022;35:100452. 10.1016/j.edurev.2021.100452

[ref4] Ary D Jacobs LC Irvine CKS : *Introduction to research in education (10th edision).* Cengage Learning;2020.

[ref5] Balt M Börnert-Ringleb M Orbach L : Reducing Math Anxiety in School Children: A Systematic Review of Intervention Research. *Frontiers in Education.* Frontiers Media S.A.;2022; Vol.7. 10.3389/feduc.2022.798516

[ref6] BERA: *Ethical guidelines for educational research.* British Educational Research Association; 4th ed. 2018.

[ref7] Bertrand MG Sezer HB Namukasa IK : Exploring AR and VR Tools in Mathematics Education through Culturally Responsive Pedagogies. *Digit. Exp. Math. Educ.* 2024;10(3):462–486. 39497730 10.1007/s40751-024-00152-x PMC11533874

[ref8] Bower M : *Design of technology-enhanced learning: Integrating research and practice.* Emerald Publishing;2019.

[ref9] Braun V Clarke V : Reflecting on reflexive thematic analysis. *Qual. Res. Sport, Exerc. Health.* 2019;11(4):589–597. 10.1080/2159676X.2019.1628806

[ref10] Bulut M Ferri RB : A systematic literature review on augmented reality in mathematics education. *Eur. J. Sci. Math. Educ.* 2023; Vol.11(3): pp.556–572. Bastas. 10.30935/scimath/13124

[ref11] Canright JP White Brahmia S : Modeling novel physics in virtual reality labs: An affective analysis of student learning. Physical Review. *Phys. Educ. Res.* 2024;20(1). 10.1103/PhysRevPhysEducRes.20.010146

[ref12] Carey E Devine A Hill F : The chicken or the egg? The direction of the relationship between mathematics anxiety and mathematics performance. *Front. Psychol.* 2019;10:1613. 26779093 10.3389/fpsyg.2019.01613 PMC4703847

[ref13] Chen C Gao Y Xie H : Augmented reality applications for mathematical creativity: A systematic review. *J. Comput. Educ.* 2023;10(3):627–646. 10.1007/s40692-023-00287-7

[ref14] Chen YC : Effect of Mobile Augmented Reality on Learning Performance, Motivation, and Math Anxiety in a Math Course. *J. Educ. Comput. Res.* 2019;57(7):1695–1722. 10.1177/0735633119854036

[ref15] Crandall R Karadoğan E : Designing pedagogically effective haptic systems for learning: A review. *Applied Sciences (Switzerland).* 2021;11(14). 10.3390/app11146245

[ref16] Creswell JW Guetterman TC : *Educational research: Planning, conducting, and evaluating quantitative and qualitative research.* Pearson; 6th ed. 2019.

[ref17] Debrenti E : Game-Based Learning experiences in primary mathematics education. *Frontiers in Education.* 2024;9. 10.3389/feduc.2024.1331312

[ref18] Depascale M Butler LP Ramani GB : The Relation Between Math Anxiety and Play Behaviors in 4-to 6-Year-Old Children. *Journal of Numerical Cognition.* 2023;9(1):89–106. 10.5964/jnc.9721

[ref19] Erbaş AK Demirer V : Impact of augmented reality technology on geometry skills and motivation of preschool children. *Educ. Inf. Technol.* 2025. 10.1007/s10639-025-13631-4

[ref20] Ersozlu Z : The role of technology in reducing mathematics anxiety in primary school students. *Contemp. Educ. Technol.* 2024; Vol.16(3). Bastas. 10.30935/cedtech/14717

[ref21] Etikan I Bala K : Sampling and sampling methods. *Biometrics & Biostatistics International Journal.* 2017;5(6):00149. 10.15406/bbij.2017.05.00149

[ref22] Field A : *Discovering statistics using IBM SPSS statistics.* Sage; 6th ed. 2021.

[ref23] Fredricks JA Blumenfeld PC Paris AH : School engagement: Potential of the concept, state of the evidence. *Rev. Educ. Res.* 2016;74(1):59–109. 10.3102/00346543074001059

[ref24] Garzón J Kinshuk Baldiris S : How do pedagogical approaches affect the impact of augmented reality on education? A meta-analysis and research synthesis. *Educational Research Review.* Elsevier Ltd.;2020; Vol.31:100334. 10.1016/j.edurev.2020.100334

[ref25] Hamad J Bianchi M Ferrari V : Integrated Haptic Feedback with Augmented Reality to Improve Pinching and Fine Moving of Objects. *Applied Sciences (Switzerland).* 2025;15(13). 10.3390/app15137619

[ref26] Hanggara Y Qohar A Sukoriyanto : The Impact of Augmented Reality-Based Mathematics Learning Games on Students’ Critical Thinking Skills. *International Journal of Interactive Mobile Technologies.* 2024;18(7):173–187. 10.3991/ijim.v18i07.48067

[ref27] Hatira A Sarac M : Touch to Learn: A Review of Haptic Technology’s Impact on Skill Development and Enhancing Learning Abilities for Children. *Advanced Intelligent Systems.* John Wiley and Sons Inc.;2024;6(6). 10.1002/aisy.202300731

[ref28] Hatira H : Touch to learn: A review of haptic technology’s impact on skill development and enhancing learning abilities for children. *Adv. Intell. Syst.* 2024;6(4):2300731. 10.1002/aisy.202300731

[ref29] Ibáñez MB Delgado-Kloos C : Augmented reality for STEM learning: A systematic review. *Comput. Educ.* 2018;123:109–123. 10.1016/j.compedu.2018.05.002

[ref30] Jannah M Halim A Asmara L : Analisis kecemasan matematika siswa sekolah dasar. *Jurnal Ilmiah Pendidikan Dasar.* 2024;9(1):33–41. Reference Source

[ref31] Jiang H Zhu D Chugh R : Virtual reality and augmented reality-supported K-12 STEM learning: trends, advantages and challenges. *Educ. Inf. Technol.* 2025;30(9):12827–12863. 10.1007/s10639-024-13210-z

[ref32] Johnson RB Onwuegbuzie AJ : Mixed methods research: A paradigm whose time has come. *Educ. Res.* 2020;33(7):14–26. 10.3102/0013189X033007014

[ref33] Kahveci EN : Game-Based Learning and Math Anxiety: An Evidence-Based Framework For Math Anxiety Reduction In Digital Learning Games And Its Application In Game Analysis And Design. 2022. 10.13140/RG.2.2.11342.06720

[ref34] Kaźmierczak R Grunwald G Skowroński R : Augmented reality tools for mathematics and geoscience education. *Sci. Rep.* 2025;15(1):17129. 40382466 10.1038/s41598-025-02090-z PMC12085572

[ref35] Koivisto J Hamari J : The rise of motivational information systems: A review of gamification research. *Int. J. Inf. Manag.* 2019;45:191–210. 10.1016/j.ijinfomgt.2018.10.013

[ref36] Lagos San Martín N Ossa C Kaur Swaran Singh C : Influence of game-based learning in mathematics education on the students’ cognitive and affective domain: A systematic review. *Front. Psychol.* 2023;1. Reference Source 10.3389/fpsyg.2023.1105806 PMC10086333 37057144

[ref37] Li M Wang L Zhang Y : Effects of AR mathematical picture books on primary school students’ geometric thinking, cognitive load and flow experience. *Educ. Inf. Technol.* 2024;29:24627–24652. 10.1007/s10639-024-12768-y

[ref38] Lincoln YS Guba EG : *Naturalistic inquiry.* Sage; 2nd ed. 2018.

[ref39] Mangkuwibawa A Rohman A Hidayati L : The relationship between math anxiety and numerical performance among elementary students. *Mudarrisa: Jurnal Kajian Pendidikan Islam.* 2023;15(1):45–60. Reference Source

[ref40] Marichal S Rosales A González Perilli F : Auditory and haptic feedback to train basic mathematical skills of children with visual impairments. *Behav. Inform. Technol.* 2023;42(8):1081–1109. 10.1080/0144929X.2022.2060860

[ref41] Miles MB Huberman AM Saldaña J : *Qualitative data analysis: A methods sourcebook.* Sage; 4th ed. 2020.

[ref42] Mirza T Dutta R Tuli N : Leveraging augmented reality in education involving new pedagogies with emerging societal relevance. *Discover Sustainability.* Springer Nature;2025; Vol.6(1). 10.1007/s43621-025-00877-8

[ref43] Mitchell L George L : Exploring mathematics anxiety among primary school students: Prevalence, mathematics performance and gender. *Int. Elect. J. Math. Educ.* 2022;17(3):em0692. 10.29333/iejme/12073

[ref44] Möhring W Moll L Szubielska M : Mathematics anxiety and math achievement in primary school children: Testing different theoretical accounts. *J. Exp. Child Psychol.* 2024;247:106038. 39137504 10.1016/j.jecp.2024.106038

[ref45] Moreno R Khodabandeh R Rahimi A : Development of AR-based educational games for mathematics learning in elementary schools. *Education and Information Technologies. Advance online publication.* 2023. 10.1007/s10639-023-12115-8

[ref46] Mutis I Oberemok M : Haptic Technology Interaction Framework in Engineering Learning: A Taxonomical Conceptualization. *Computer Applications in Engineering Education.* John Wiley and Sons Inc.;2025; Vol.33(2). 10.1002/cae.70009

[ref47] Nur A : The effect of math anxiety on numeracy literacy of elementary school students. *Jurnal Ilmu Edukasi.* 2024;6(2):115–124. Reference Source

[ref48] O’Hara G Kennedy H Naoufal M : The role of the classroom learning environment in students’ mathematics anxiety: A scoping review. *Br. J. Educ. Psychol.* 2022;92(4):1458–1486. 35538624 10.1111/bjep.12510

[ref49] Pahmi S Hendriyanto A Sahara S : Assessing the Influence of Augmented Reality in Mathematics Education: A Systematic Literature Review. *International Journal of Learning, Teaching and Educational Research.* 2023;22(5):1–25. 10.26803/ijlter.22.5.1

[ref50] Pekrun R : Emotions and learning. Mayer R Alexander P , editors. *Handbook of Research on Learning and Instruction.* Routledge; 2nd ed. 2021; pp.327–348.

[ref51] Pila S Piper AM Lauricella A : Preschoolers’ stem learning on a haptic enabled tablet. *Multimodal Technologies and Interaction.* 2020;4(4):1–25. 10.3390/mti4040087

[ref52] Pizzie RG Kraemer DJM : Strategies for remediating the impact of math anxiety on high school math performance. *Npj Science of Learning.* 2023;8(1):44. 37779133 10.1038/s41539-023-00188-5 PMC10543627

[ref53] Polydoros G Galitskaya V Pergantis P : Innovative AI-Driven Approaches to Mitigate Math Anxiety and Enhance Resilience Among Students with Persistently Low Performance in Mathematics. *Psychology International.* 2025;7(2):46. 10.3390/psycholint7020046

[ref54] Putra RA : Factors influencing mathematics anxiety among elementary school students in Pacitan. *Proceedings of International Conference on Learning and Education (ICLE).* 2024;2(1):77–85. Reference Source

[ref55] Radišić J Peixoto F Caetano T : Scared, Bored or Happy? Latent Profile Analyses of Primary School Students’. *Academic Emotions about Math. Education Sciences.* 2024;14(8). 10.3390/educsci14080841

[ref56] Radu I : Augmented reality in education: A meta-review and cross-media analysis. *Comput. Educ.* 2021;171:104225. 10.1016/j.compedu.2021.104225

[ref57] Richard G Pietrzak T Argelaguet Sanz F : Studying the Role of Haptic Feedback on Virtual Embodiment in a Drawing Task. *Virtual Reality.* 2021;1:573167. 10.3389/frvir.2020.573167i Reference Source

[ref58] Sammallahti E Finell J Jonsson B : A Meta-Analysis of Math Anxiety Interventions. *Journal of Numerical Cognition.* 2023;9(2):346–362. 10.5964/jnc.8401

[ref59] Santos MEC Chen A Taketomi T : Augmented reality learning experiences: Survey of prototype design and evaluation. *IEEE Trans. Learn. Technol.* 2020;13(3):514–531. 10.1109/TLT.2020.2978270

[ref60] Schoenherr J Schukajlow S Pekrun R : Emotions in mathematics learning: a systematic review and meta-analysis. *ZDM - Mathematics Education.* 2025;57(4):603–620. 10.1007/s11858-025-01651-w

[ref61] Schutera S Schnierle M Wu M : On the potential of augmented reality for mathematics teaching with the application cleARmaths. *Educ. Sci.* 2021;11(8). 10.3390/educsci11080368

[ref62] Sugiarti R Kusmayanti D : Mathematics anxiety of elementary school students during COVID-19 pandemic. *Jurnal Pendidikan Dasar.* 2022;13(2):123–134. Reference Source

[ref63] Supriadi N Wan Jamaluddin Z Suherman S : The role of learning anxiety and mathematical reasoning as predictor of promoting learning motivation: The mediating role of mathematical problem solving. *Think. Skills Creat.* 2024;52:101497. 10.1016/j.tsc.2024.101497

[ref64] Tanggu Mara A : List of Figures and Tables - Bringing Numbers to Life and Reducing Anxiety: An Augmented Reality and Haptic Feedback-Based Mathematics Game for Primary School Students.[Data set]. *Zenodo.* 2025a. 10.5281/zenodo.17120556

[ref65] Tanggu Mara A : Letter of Permission - Bringing Numbers to Life and Reducing Anxiety: An Augmented Reality and Haptic Feedback-Based Mathematics Game for Primary School Students. *Zenodo.* 2025b. 10.5281/zenodo.17120705

[ref66] Uğraş H : Research on mathematics anxiety in primary school: bibliometric analysis and evaluation of trends. *Frontiers in Psychology.* Frontiers Media SA;2025; Vol.16. 10.3389/fpsyg.2025.1545556 PMC12369395 40851609

[ref67] Vanbecelaere S Cornillie F Sasanguie D : The effectiveness of an adaptive digital educational game for the training of early numerical abilities in terms of cognitive, noncognitive and efficiency outcomes. *Br. J. Educ. Technol.* 2021;52(1):112–124. 10.1111/bjet.12957

[ref68] Vankúš P : Influence of game-based learning in mathematics education on students’ affective domain: A systematic review. *Mathematics.* MDPI AG;2021; Vol.9(9). 10.3390/math9090986 PMC10086333 37057144

[ref69] Volioti C Orovas C Sapounidis T : Augmented Reality in Primary Education: An Active Learning Approach in Mathematics. *Computers.* 2023;12(10). 10.3390/computers12100207

[ref70] Wang C Li X Wang HJ : The mediating effect of math self-efficacy on the relationship between parenting style and math anxiety. *Front. Psychol.* 2023;14. 37359871 10.3389/fpsyg.2023.1197170 PMC10289032

[ref71] Wang J Lindeman RW Lee GA : Haptic feedback for augmented reality: State-of-the-art and future directions. *Frontiers in Virtual Reality.* 2021;2:20. 10.3389/frvir.2021.666695

[ref72] Wangid MN Rudyanto HE Gunartati. : The Use of AR-assisted storybook to reduce mathematical anxiety on elementary school students. *International Journal of Interactive Mobile Technologies.* 2020;14(6):195–204. 10.3991/IJIM.V14I06.12285

[ref73] Wu J Jiang H Long L : Effects of AR mathematical picture books on primary school students’ geometric thinking, cognitive load and flow experience. *Educ. Inf. Technol.* 2024;29(18):24627–24652. 10.1007/s10639-024-12768-y

[ref74] Yu S Liu Q Ma J : Applying augmented reality to enhance physics laboratory experience: does learning anxiety matter? *Interact. Learn. Environ.* 2023;31(10):6952–6967. 10.1080/10494820.2022.2057547

[ref75] Zapata M Ramos-Galarza C Valencia-Aragón K : Enhancing mathematics learning with 3D augmented reality escape room. *International Journal of Educational Research Open.* 2024;7:100389. 10.1016/j.ijedro.2024.100389

[ref76] Zhang Y Li H Chen J : Haptic feedback in education: Enhancing learning through multisensory interaction. *Comput. Educ.* 2023;192:104672. 10.1016/j.compedu.2023.104672

