# Supplementary material for: Bringing Numbers to Life and Reducing Anxiety: An Augmented Reality and Haptic Feedback-Based Mathematics Game for Primary School Students
Source: F1000Res. 2026 Mar 25;14:1131. Originally published 2025 Oct 17. [Version 2] doi: 10.12688/f1000research.170339.2 (PMC13673076; doi:10.12688/f1000research.170339.2)
Supplement: Peer review history

# Reviewer report: Reviewer response for version 2

**DOI:** 10.5256/f1000research.192984.r528693

**Published:** 2026-10-15

**Authors:** Daniel Jesayanto Jaya

**Affiliations:** 1 Universitas Negeri Jakarta, East Jakarta, Special Capital Region of Jakarta, Indonesia

I have read version 2 and considered the published reviewer reports and the authors’ responses concerning version 1. The revision improves the positioning of the study as a proof of concept, expands the theoretical discussion, and more explicitly acknowledges potential confounding factors and the inability to isolate the contributions of AR and haptic feedback. I consider the revised article acceptable overall and have selected Approved. Nevertheless, several remaining inconsistencies should be addressed to improve the accuracy and transparency of the published record.

1. Please reconcile the conceptual understanding scores in Tables 1 and 3. Table 1 reports experimental-group means of 59 and 82 and control-group means of 58 and 62, whereas Table 3 reports experimental-group means of 53.25 and 73.22 and control-group means of 54.43 and 62.43. If these represent different measures, samples, or scoring procedures, please explain this explicitly. Otherwise, please correct the tables and all corresponding statements, including the abstract and effect-size calculations.
2. Table 2 includes Cohen’s d values for within-group pre–post comparisons, although its note and accompanying text state that effect sizes are reported only for the between-group post-test comparison. Please make the table and explanation consistent and specify the calculation used for each retained effect size.
3. Please clarify the analyses underlying Table 4, particularly the error degrees of freedom of 39 and 38 in relation to the stated sample of 120 students. Identify the sample size and statistical model for each analysis, explain any exclusions or missing data, and specify whether the reported η² values represent eta squared or partial eta squared. The ANCOVA and MANOVA mentioned in the methods should also be reported sufficiently to permit evaluation of their results.
4. The response to the previous review describes additional information on stratified sampling, baseline equivalence, intervention fidelity, and instrument validity. Please ensure that these details are explicitly documented in the manuscript or clearly identified supplementary materials. Further information about the haptic hardware and its integration with the game would also support replication.
5. Please conduct a final editorial check. In particular, the unrelated sentence beginning “Al-i-Imran (240)” in Section 3.1 should be removed or corrected. The anxiety scores reported on a five-point scale should be clearly related to the MARS-E scores in Table 3, and the remaining causal or mechanistic statements should be aligned with the acknowledged limitations of the quasi-experimental design.

These comments are intended to strengthen reporting precision and reproducibility while recognizing the improvements made in version 2 and the potential educational value of the intervention.

Is the work clearly and accurately presented and does it cite the current literature?

Partly

If applicable, is the statistical analysis and its interpretation appropriate?

Partly

Are all the source data underlying the results available to ensure full reproducibility?

Partly

Is the study design appropriate and is the work technically sound?

Partly

Are the conclusions drawn adequately supported by the results?

Partly

Are sufficient details of methods and analysis provided to allow replication by others?

Partly

Reviewer Expertise:

Technical and vocational education and training (TVET); technology-enhanced learning; virtual reality in education; learning anxiety; career transformation and entrepreneurship; and qualitative and mixed-methods research.

I confirm that I have read this submission and believe that I have an appropriate level of expertise to confirm that it is of an acceptable scientific standard.

# Reviewer report: Reviewer response for version 1

**DOI:** 10.5256/f1000research.187783.r428954

**Published:** 2025-12-04

**Authors:** Edith Debrenti

**Affiliations:** 1 Partium Christian University, Oradea, Romania

This study developed a learning model integrating augmented reality (AR) and haptic feedback to create an interactive and multisensory mathematics experience. A quasi-experimental design was conducted with 120 students divided into experimental and control groups. The experimental group used the AR–haptic mathematics game for six weeks, while the control group learned through textbooks and teacher explanations. Data was gathered by using the conceptual understanding tests, a mathematics anxiety scale, classroom observations and student interviews. The quantitative data were analyzed by t-test, ANCOVA and MANOVA analysis; qualitative data were analyzed positionally. The results indicated significant progress in experimental groups’ conceptual understanding and mathematics anxiety decrease when compared to the control group.

 Researchers found that the integration of AR and haptic feedback effectively enhanced both cognitive and affective outcomes in mathematics learning. The multisensory environment enabled students to interact with abstract concepts in tangible ways, reducing anxiety and promoting positive attitudes toward mathematics. This provides a proof of concept for the effectiveness of immersive technologies as assistive tools in the enhancement of academic achievement and emotional health related to elementary level mathematics learning.

 This research provides the following contributions to technology-enhanced learning: (1) combining AR and haptic feedback into a single mathematics game developed for elementary school students, (2) targeting both cognitive outcomes and affective issues such as math anxiety, and (3) operationalizing the principles of embodied design in a game-based learning environment to promote sustainable, emotionally positive learning of mathematics.

 My comments:

 1.„There was significant difference between experimental and control

 group after the pretest level (Mean Difference = 23, p = 0.001), indicating that the intervention had an impact on the team members’ learning achievement”.

**It is wrong: this is the pre-test versus post-test difference in case of experimental group.**

 2. Table 2 is incomplete: missing values. What does „nan” mean?

 3. To correct: (M1C.62.43,SD1C.9.42).

 4. To correct: Tabel 3: „Eksperimen”, „Kontrol”

 5.One of the most surprising results was that the mathematical anxiety of participants in both groups decreased, even in the control group. **How do you explain this?**

 „Only minimal decreases in math anxiety were found in the control group”. From 66.7 to 56.25 it is not so minimal.

 6. To correct in Final remark: ... to the emerging **technolgoy**-enhanced...

 7. When the reader first encounters VR, MA, CUT, MARS-E, and FGD in the text, it is necessary to explain what these abbreviations stand for.

 8. The authors did not adequately process the results of the qualitative study; they should have incorporated them more thoroughly into the article.

 9. I think that one of the limitations of the research is that the results cannot be separated, we cannot tell which component (visual AR vs tactile feedback) accounted for the observed effects. This must also be entered in the Limitations section.

 10. In case of references: several links do not work, articles are not available, e.g.

 Santos MEC, Chen A, Taketomi T, *et al.*: Augmented reality learning experiences: Survey of prototype design and evaluation. *IEEE Trans. Learn. Technol.* 2020; **13**(3): 514–531.

 Congratulations on the article! It addresses an important topic, presents original findings, and makes a valuable contribution to the field.

Is the work clearly and accurately presented and does it cite the current literature?

Yes

If applicable, is the statistical analysis and its interpretation appropriate?

I cannot comment. A qualified statistician is required.

Are all the source data underlying the results available to ensure full reproducibility?

Yes

Is the study design appropriate and is the work technically sound?

Yes

Are the conclusions drawn adequately supported by the results?

Partly

Are sufficient details of methods and analysis provided to allow replication by others?

Partly

Reviewer Expertise:

Mathematical educationTeaching ExperienceTeacher EducationResearch MethodologyEducation ResearchHigher Education ResearchSTEMSTEAMGBLDGBL

I confirm that I have read this submission and believe that I have an appropriate level of expertise to confirm that it is of an acceptable scientific standard.

# Reviewer report: Reviewer response for version 1

**DOI:** 10.5256/f1000research.187783.r428951

**Published:** 2025-11-06

**Authors:** Farouq Sessah Mensah

**Affiliations:** 1 Stockholm University, Stockholm, Sweden

**1. Overall Evaluation**

 This paper presents an ambitious attempt to integrate augmented reality (AR) and haptic feedback into a mathematics game designed to enhance conceptual understanding and reduce mathematics anxiety among primary school students. While the study demonstrates innovative thinking and solid intentions, several weaknesses in methodological rigour **, **theoretical grounding, and reporting clarity undermine its contribution. The work is potentially publishable but requires substantial revision to meet the standards expected of peer-reviewed educational technology research.

**2. Significance and Originality**

**Strengths:**

- The combination of AR and haptic feedback for primary mathematics learning is novel and relevant.
- The study’s dual focus on cognitive (conceptual understanding) and affective (anxiety) outcomes aligns with current research priorities in technology-enhanced learning.
- The development of a fully implemented prototype, *Kidar Playtime*, demonstrates commendable applied effort.

**Limitations:**

- The manuscript’s claim to originality is overstated. Recent studies (e.g., Hatira & Sarac, 2024; Mutis & Oberemok, 2025; Moreno et al., 2023) have already examined multimodal AR or haptic interventions in early STEM contexts.
- The conceptual contribution to embodied learning theory remains superficial. The authors invoke “embodied design” and “multisensory learning” without a clear theoretical articulation or framework for how these constructs inform the intervention’s pedagogical model.
- There is little discussion of cultural or contextual factors (e.g., curriculum) that could situate the study more meaningfully within its educational environment.

**Recommendation:** Reframe the originality claim as a *proof of concept* rather than a theoretical innovation. Deepen engagement with embodied cognition literature beyond surface citation.

**3. Methodology**

**Strengths:**

- The use of a quasi-experimental design with control and experimental groups is appropriate for intervention-based research.
- Ethical procedures and institutional permissions are clearly documented.
- Mixed-method triangulation (tests, surveys, interviews, observation) strengthens internal validity.

**Weaknesses and concerns:**

- **Randomization and equivalence:** The “stratified random sampling” lacks procedural detail. It is unclear how the groups were balanced on prior performance or anxiety levels. Without random assignment, causality remains tentative.
- **Intervention fidelity:** Although teachers received two weeks of training, the paper does not describe monitoring of fidelity during implementation—an important issue in educational technology trials.
- **Measurement instruments:** The *Conceptual Understanding Test* (CUT) and *MARS-E* are reported with Cronbach’s alphas, but no evidence of construct validity or item examples are provided. The local adaptation of MARS-E lacks psychometric justification.
- **Statistical reporting:**
  - Tables contain inconsistencies (e.g., missing standard deviations, co-efficient of variation, “nan” values in Table 2).
  - ANCOVA and MANOVA results are mentioned but not systematically presented with F, df, and p-values for all variables.
  - Effect sizes (Cohen’s *d* = 2.35) are unusually large and warrant scrutiny; such a large value suggests possible measurement artefacts or overfitting.
- **Qualitative data:** Thematic analysis is mentioned but minimally reported. Only a few student quotes are presented, with no analytic depth or link to the quantitative trends.

**Recommendation:** Provide transparent statistical tables with full reporting standards (APA/CONSORT). Clarify sampling and validity procedures. Deepen qualitative interpretation beyond anecdotal quotations.

**4. Results and Interpretation**

**Strengths:**

- The results clearly indicate improvement in both achievement and reduced anxiety in the experimental group.
- The integration of quantitative and qualitative results shows a positive direction for mixed-methods synthesis.

**Weaknesses:**

- The discussion section conflates results and interpretation, leading to redundancy and descriptive narrative rather than analytical insight.
- Causal language (“the intervention caused significant improvement”) is too strong given the quasi-experimental design.
- The statistical claims (e.g., *p* = 0.001, *d* = 2.35) are not contextualized within practical classroom effect sizes.
- The paper fails to critically engage with possible confounding variables such as novelty effect, teacher enthusiasm, or increased attention from researchers all of which may have contributed to gains.
- The integration of AR and haptics is not analytically disentangled; we cannot tell which component (visual AR vs tactile feedback) accounted for the observed effects.

**Recommendation:** Reorganize this section to separate descriptive results from interpretative claims. Include limitations of inference and consider mediation analysis or post-hoc exploration of differential effects.

**5. Theoretical Framing and Discussion**

**Strengths:**

- References to embodied cognition, constructivism, and affective engagement are timely and relevant.
- The study’s aim to bridge cognitive and affective dimensions aligns with emerging frameworks in affect-aware learning technologies.

**Weaknesses:**

- The theoretical integration is weak. “Embodied learning” and “affect-aware technologies” are invoked rhetorically rather than analytically operationalized.
- The discussion lacks mechanistic explanation, that is, how precisely does haptic interaction reduce anxiety? What cognitive or neuropsychological pathways are proposed?
- The manuscript cites an overwhelming number of secondary sources but does not critically synthesize them. The literature review reads as an annotated catalogue rather than a conceptual argument.
- The discussion does not connect findings to teacher practice, curriculum design, or pedagogical affordances of AR-haptic learning in the real world.

**Recommendation:** Reframe the discussion to engage critically with embodied cognition models (e.g., Abrahamson et al., 2020) and cognitive-affective coupling frameworks (e.g., Pekrun, 2021). Avoid excessive citation without synthesis.

**6. Presentation and Style**

**Strengths:**

- The manuscript is well-organized into clear subsections.
- Ethical transparency and data sharing (Zenodo link) enhance reproducibility.

**Weaknesses:**

- Numerous language and grammar issues (e.g., “math is gets associated,” “vertigo directly affects your numeracy”) compromise readability.
- Figures and tables are not well-integrated into the narrative—many are “Supplementary,” which obscures key evidence.
- Some references are inconsistent or incorrectly formatted, e.g., “Hatirassa & Saracss, 2024,” “Kaźmierczak et al., Skowroński, Kaźmierczak, & Kowalczyk-Page 8-572.”
- The writing style occasionally drifts into advocacy rather than maintaining analytical objectivity.

**Recommendation:** Substantially edit for clarity, concision, and grammatical precision. Reorganize tables and figures into the main text for readability. Apply a consistent referencing style.

**7. Limitations and Future Directions**

 The authors acknowledge sample size and duration as limitations, but do not address technological limitations (e.g., hardware calibration, haptic glove precision) or pedagogical transferability (teacher training, classroom management). Future research should explore longitudinal retention effects, cost-benefit analyses, and comparative studies isolating AR versus haptic influences.

**8. Recommendation**

**Decision:** Major Revision

 The manuscript has **potential** due to its innovative combination of AR and haptic technologies, but it currently reads as an overextended proof-of-concept study lacking theoretical depth, statistical transparency, and linguistic refinement. With rigorous methodological clarification, tighter theoretical articulation, and improved writing, this paper could make a meaningful contribution to research on embodied and affect-aware learning technologies in mathematics education.

Is the work clearly and accurately presented and does it cite the current literature?

Partly

If applicable, is the statistical analysis and its interpretation appropriate?

Partly

Are all the source data underlying the results available to ensure full reproducibility?

Partly

Is the study design appropriate and is the work technically sound?

Partly

Are the conclusions drawn adequately supported by the results?

Partly

Are sufficient details of methods and analysis provided to allow replication by others?

Partly

Reviewer Expertise:

Didactics of mathematics, instructional technology, practiced-based teacher education,

I confirm that I have read this submission and believe that I have an appropriate level of expertise to confirm that it is of an acceptable scientific standard, however I have significant reservations, as outlined above.

# Response

**Published:** 2025-12-04

**Authors:** Andry Ananda Putra Tanggu Mara andryananda.2023

Dear Reviewer,

 We would like to thank you for your thorough and constructive feedback. Your comments have significantly improved the clarity, methodological rigor, and theoretical contribution of our manuscript. Below, we provide a point-by-point response addressing every issue raised, followed by the revisions incorporated into the manuscript.

 We agree with this assessment and have revised the introduction, contribution statement, and theoretical sections accordingly. Revisions Made:

1. Reframed contribution explicitly as a **proof of concept**, not a theoretical innovation.
2. Added a deeper synthesis of embodied cognition frameworks (Abrahamson et al., 2020; Nemirovsky et al., 2010) and clarified how sensorimotor grounding informed the design principles.
3. Discussed cognitive–affective coupling (Pekrun, 2021) to explain interactions between conceptual understanding and anxiety reduction.
4. Added contextual justification referencing the Indonesian mathematics curriculum and socio-educational conditions.

We appreciate these concerns and have enhanced methodological transparency. Revisions Made:

1. Added detailed explanation of stratified random sampling, including baseline equivalence testing with pre-test t-tests.
2. Included description of intervention fidelity monitoring: fidelity checklists, teacher logs, and observation cycles.
3. Added construct validity evidence for CUT and adapted MARS-E, including expert review, item examples, and pilot factor alignment.
4. Re-structured statistical tables according to APA/CONSORT standards; removed “nan” values; added complete F, df, p, and η² where relevant.
5. Explained the large effect size cautiously and noted the possibility of novelty effects or measurement properties.
6. Expanded qualitative results: added thematic structure, analytic procedures, multiple quotes, and links to quantitative trends.

We agree and have reorganized the Results and Discussion sections. **Revisions Made:**

1. Results (Section 3) now contain **only descriptive statistics**, with no interpretative statements.
2. Interpretation (Section 4) is structurally separated and explicitly uses **non-causal language** (e.g., “associated with” rather than “caused”).
3. Added contextual discussion comparing effect sizes with those in prior educational technology research.
4. Added a dedicated paragraph addressing **potential confounders** (novelty effect, teacher enthusiasm, researcher presence, time-on-task).
5. Clarified that the design does not allow separation of AR vs. haptic contributions; recommended **dismantling studies** in future research.

We revised Section 4 extensively to integrate theoretical models meaningfully. **Revisions Made:**

1. Added analysis based on **embodied cognition mechanisms**, including sensorimotor grounding, perceptual–motor coordination, and action-based conceptualization.
2. Integrated **Pekrun’s Control–Value Theory**, explaining how reduced anxiety and perceived control interact with conceptual learning.
3. Synthesized both theoretical lenses into an integrated interpretive model of cognitive–affective embodiment.

We appreciate the need for clearer academic writing. **Revisions Made:**

1. Conducted a full **language and grammar revision**, removing awkward syntax and improving clarity.
2. Integrated key tables and figures directly into the manuscript instead of placing them solely in supplementary materials.
3. Standardized all references according to journal guidelines, correcting names, spellings, and formatting.
4. Revised prose to maintain **analytical neutrality**, avoiding promotional or advocacy tone.

We sincerely thank the reviewer for the thoughtful and rigorous feedback. The revisions have substantially improved the clarity, theoretical depth, and methodological robustness of the manuscript. We hope the updated version now meets the publication standards and expectations of the journal.

 Thank You

 On behalf of all authors,

 Andry Tanggu Mara
